# Eosinophilic Metaplasia in the Epididymis in Association With Lipofuscin Pigment: A Case Report

**DOI:** 10.7759/cureus.34961

**Published:** 2023-02-14

**Authors:** Elena I Gerakova

**Affiliations:** 1 Department of General and Clinical Pathology, Forensic Medicine and Deontology, Medical University of Plovdiv, Plovdiv, BGR

**Keywords:** cytoplasmic granules in epididymis, lipofuscin deposits in epididymis, ductal obstruction, eosinophilic metaplasia, epididymis

## Abstract

We present the case of a 54-year-old man with a cystic formation measuring 0.6 cm in the head of the epididymis. Histologically, the lesion showed intense granular eosinophilic transformation of the cytoplasm. The finding was assessed as eosinophilic metaplasia (EM) and showed association with and deposition of lipofuscin pigment. The EM in the epididymis presents a sign of intracytoplasmic lysosomal accumulation, which serves as a microscopic indicator of ductal obstruction. The presented unique finding was compared with the data reported in the literature. In this case report, we describe an extremely rare metaplastic lesion in the epididymis.

## Introduction

Metaplasia is a process of transformation of one tissue into another related to the first (initial) one [1]. Metaplasia may be part of a normal maturation process or caused by an abnormal stimulus. For such conversion to be possible, tissues must arise from the same embryonic layer.

According to our literature review, there are several reports of eosinophilic metaplasia (EM) in the epididymal epithelial cells [2,3]. Studies have referred to them as Paneth cell-like changes (PCLC) or granular changes and are found in the benign epididymal epithelium [2,3]. Here, a case of EM in a benign cystic epididymal epithelium is presented. From the cases we found in the literature, epididymal EM is not described in the wall of benign cystic formations.

## Case presentation

A 54-year-old male presented with a cystic formation in the head of the epididymis. A cystic formation with a diameter of 0.6 cm was observed. It was localized in the head of the epididymis, which clinically caused discomfort for the patient but without micturition disorders. Ultrasound examination excluded malignancy. A digital rectal examination showed no changes in the prostate, which measured between 20 and 30 g.

**Figure 1:**
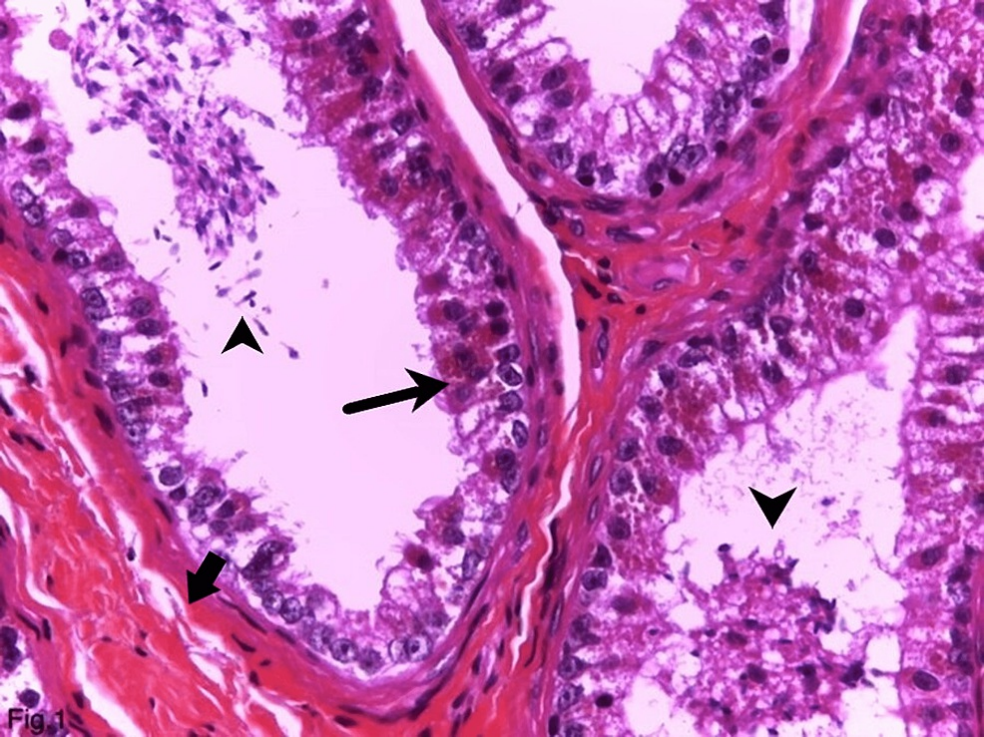
Eosinophilic metaplasia in the benign epididymal epithelium (hematoxylin and eosin, ×400). Thin arrow: Benign epithelial cells in the wall of cystic dilated ducts, with abundant cytoplasm, filled with eosinophilic cytoplasmic granules and simultaneously containing lipofuscin granules which are smaller in size and yellow-brown. Arrowhead: Sperm cells in the ductal lumen. Thick arrow: Interductal stroma with fibrosis and hyalinosis.

**Figure 2:**
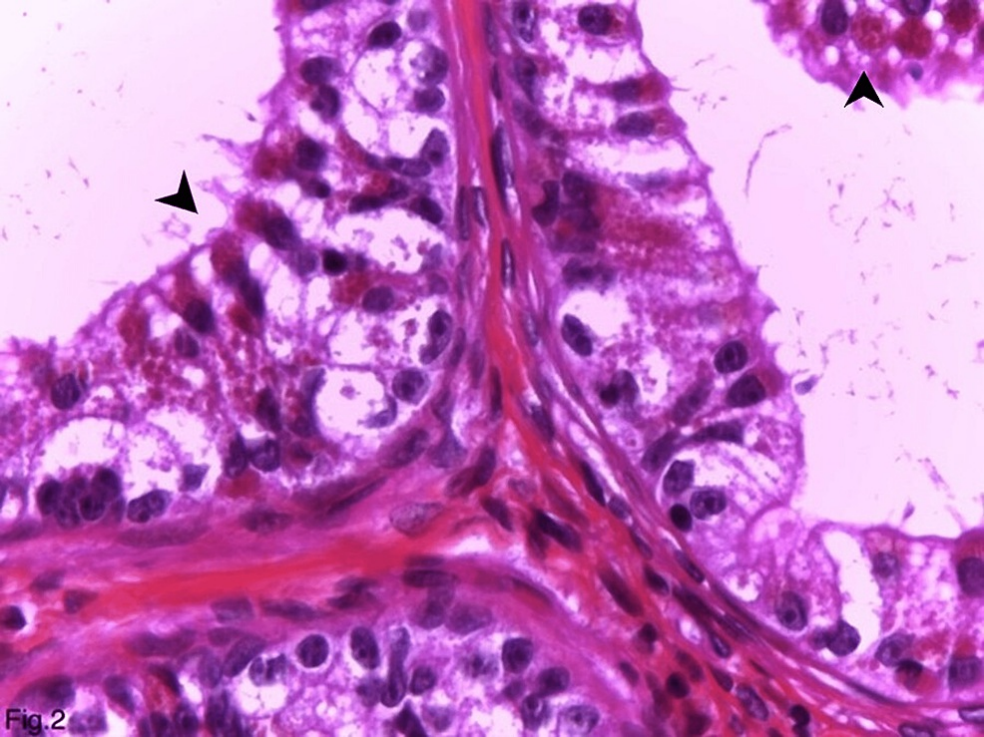
Eosinophilic metaplasia in the benign epididymal epithelium in association and transition of lipofuscin pigment (hematoxylin and eosin, ×630). Arrowhead: Eosinophilic granules and lipofuscin pigment in epithelial cells.

Macroscopically, a cystic formation with a diameter of 0.6 cm and a smooth wall was present. The histopathological investigation showed epididymal tissue, with dilated interductal stroma due to fibrosis and hyalinosis. Moderately pronounced mononuclear inflammatory infiltrate was also identified (data not shown). The walls of the cystic dilated ducts were lined with benign epithelial cells that were polygonal with abundant cytoplasm and filled with eosinophilic granules (Figure 1). The latter were spherical, measuring 2-5 µm, and took up the entire cytoplasm (Figures 1, 2).

**Figure 3:**
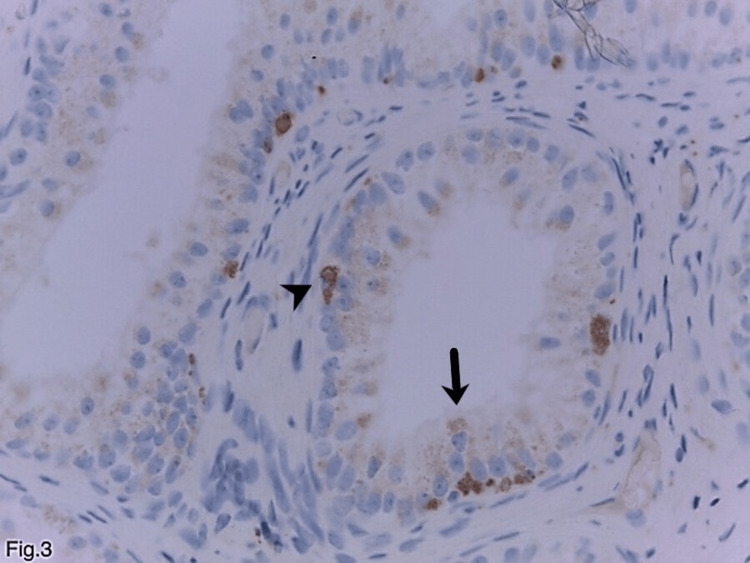
Eosinophilic metaplasia in the benign epididymal epithelium in association and transition of lipofuscin pigment; weakly positive granules, and the presence of pronounced cytoplasm staining in the subepithelial macrophages, which serve as a positive internal control staining (immunohistochemistry, anti-CD68, ×400). Thin arrow: Weakly positive granules in epithelial cells. Arrowhead: Pronounced cytoplasm staining in the subepithelial macrophages.

Along with eosinophilic granules, the cell’s cytoplasm contained typical lipofuscin granules, characterized by their smaller size and yellow-brown color on hematoxylin and eosin staining (Figure 2). The transition between the two types of granules in the same cell was frequently observed (Figure 2). No atypia was observed in the cells. They had tiny, inconspicuous nucleoli, lacked mitoses, and were localized in the center (Figure 2). Sperm cells were seen in the dilated ductal acini lumen (Figure 1). When stained with periodic acid-Schiff reaction and periodic acid-Schiff reaction with diastase, the granules showed a non-constant weak positive reaction. Immunohistochemically, the granules were positive for the lysosomal markers CD68 (Figure 3), alpha-1-antitrypsin, and IgA.

## Discussion

EM is an uncommon form of differentiation characterized by an oxyphilic appearance in individual cells. It can be very rarely observed in normal glands or never seen. It is described as varying in diameter, intensively eosinophilic granules, located in the cytoplasm and benign glandular epithelium [4,5]. It has been observed in several organs with glandular components and mucus membranes, uterine endometrium [6], prostate [4,5,7], and breast [8]. Out of all of the above-described locations, the prostate has been studied most thoroughly. In the prostate, EM is represented by secretory cytoplasmic granules with both exocrine and lysosomal characteristics [7]. They have varying diameters with predominantly ductal positioning [9]. A reliable immunohistochemical marker for the EM phenotype is MUC1, which is expressed by the granules in the cytoplasm [10]. From a pathological perspective, prostate EM is an indirect (phenotypic-type) metaplasia [7]. Normally, this process in the prostate goes along with chronic inflammation, which may be granulomatous [11] and prostate adenocarcinoma [12].

In a histopathological study of the epididymis, ЕМ (PCLC) is interpreted as intracytoplasmic lysosomal accumulation, which serves as a microscopic indicator of ductal obstruction [2]. In the prostate, the coexistence of the non-specific granulomatous prostatitis (NSGP) and EM in 100% of cases shows that NSGP, and its accompanying EM, are processes that reflect the morphological status of urinary obstruction due to benign prostate hyperplasia and the supplementary low or high-grade histologic prostatitis [11].

The eosinophilic cytoplasm changes in the epididymis (PCLC) [2] are presented as eosinophilic intracytoplasmic granules resembling those of Paneth cells in the intestinal mucosa. They are observed adjacent to benign and malignant processes in the epididymal efferent channels. Unlike true Paneth intestinal cells, the granular changes in EM cells in the epididymis do not contain phospholipase A2 [13]. Unlike EM, the true granules in Paneth cells are also negative for CD68 and anti-chymotrypsin antibodies, confirming that they are secretory vesicles.

According to some studies, the term Paneth cell-like metaplasia should be changed as there are no immunohistochemical similarities between these granules, and it is not true metaplasia [3]. They suggest that because of the accumulation of lysosomes caused by an increase in endocytic activity secondary to fluid, granular changes in the epididymal epithelial cells appear. This may not be only due to obstruction of the spermatic pathway but also due to increased secretion of fluid, e.g., testicular tumor [3].

The results in the presented case show that EM in the human epididymis is characterized by eosinophilic intracytoplasmic hyaline-like granules and globules of different stages and transition to lipofuscin pigment. They confirm, similar to the results of other authors [2], that these changes are associated with sperm pathway obstruction. In the presented case, EM was similar to that of the prostate and was seen in the benign cystic epididymal epithelium [4,5,7]. In addition, in the presented case, the association of EM and epididymal lipofuscinosis has been shown for the first time. These observations confirm the transition of EM to lipofuscin over time, which has been observed so far only in the prostate [7].

## Conclusions

EM in the human epididymis is a benign metaplastic cytoplasm change and can be a part of a cystic lesion of the organ. Obviously, similar to the prostate, mammary gland, and endometrium, EM in the human epididymis can be seen in the epithelium without the presence of a malignant process. It is possible that it can be used as a microscopic indicator for ductal obstruction. However, because it can be linked with increased accumulation of fluid, such as in testicular tumors, it is important to recognize this as a pathological entity.

The presented observation is the first detailed transition of eosinophilic cytoplasmic granules in lipofuscin pigment in the benign epididymal epithelium.
